# Evaluation of a New Cryptococcal Antigen Lateral Flow Immunoassay in Serum, Cerebrospinal Fluid and Urine for the Diagnosis of Cryptococcosis: A Meta-Analysis and Systematic Review

**DOI:** 10.1371/journal.pone.0127117

**Published:** 2015-05-14

**Authors:** Hua-Rong Huang, Li-Chao Fan, Bhavana Rajbanshi, Jin-Fu Xu

**Affiliations:** Department of Respiratory Medicine, Shanghai Pulmonary Hospital, Tongji University School of Medicine, Shanghai, China; University of Utah Health Sciences Center, UNITED STATES

## Abstract

**Background:**

A new lateral flow immunoassay (LFA) for the detection of cryptococcal antigen was developed.

**Objective:**

We aimed to systematically review all relevant studies to evaluate the diagnostic accuracy of the cryptococcal antigen LFA on serum, CSF and urine specimens.

**Methods:**

We searched public databases including PubMed, Web of Science, Elsevier Science Direct and Cochrane Library for the English-language literature published up to September 2014. We conducted meta-analyses of sensitivity, specificity, positive likelihood ratio (PLR), negative likelihood ratio (NLR) and diagnostic odds ratios (DOR) and SROC of LFA in serum and CSF, respectively. The sensitivity of LFA in urine was also analyzed. Subgroup analyses were carried out to analyze the potential heterogeneity.

**Results:**

12 studies were included in this study. The pooled sensitivity and specificity values of LFA in serum were 97.6% (95% CI, 95.6% to 98.9%) and 98.1% (95% CI, 97.4% to 98.6%), respectively. The average PLR of LFA in serum was 43.787 (95% CI, 22.60–84.81) and the NLR was 0.03 (95% CI, 0.01–0.09). The pooled DOR was 2180.30 (95% CI, 868.92–5471.00) and the AUC was 0.9968. The pooled sensitivity and specificity values of LFA in CSF were 98.9% (95% CI, 97.9% to 99.5%) and 98.9% (95% CI, 98.0% to 99.5%), respectively. The average PLR of LFA in serum was 48.83 (95% CI, 21.59–110.40) and the NLR was 0.02 (95% CI, 0.01–0.04). The pooled DOR was 2931.10 (95% CI, 1149.20–7475.90) and the AUC was 0.9974. The pooled sensitivity value of LFA in urine was 85.0% (95% CI, 78.7% to 90.1%)

**Conclusions:**

The study demonstrates a very high accuracy of LFA in serum and CSF for the diagnosis of cryptococcosis in patients at risk. LFA in urine can be a promising sample screening tool for early diagnosis of cryptococcosis.

## Introduction

Cryptococcosis is a worldwide distributed mycosis caused by *Cryptococcus neoformans* and *Cryptococcus gattii* [1]. *C*.*neoformans* mostly infects AIDS patients particularly in sub-Saharan Africa as well as immunocompromised group(e.g.organ transplanted patients) [2]. In contrast, *C*.*gattii* is responsible for infecting immunocompetent individuals on Vancouver Island and surrounding areas [3]. Cryptococcosis is mainly caused by inhalation of pathogens through the respiratory tract. The central nervous system infection is the main clinical manifestation of cryptococcosis, so cryptococcal meningitis or meningoencephalitis is the main reason of high fatality rate. The increasing incidence of cryptococcosis is associated with use of broad-spectrum antibiotics, HIV infection, tumor radiotherapy and chemotherapy, organ transplantation, increased use of corticosteroids and immunosuppressants.

The principle of cryptococcal antigen test is to detect cryptococcal capsular polysaccharide antigen in serum or body fluids [4]. Latex agglutination method (LA) is the most popular one which is more sensitive than culture. However, LA takes much labor and the judgment results may be subjective. In order to make up with the drawbacks of LA, some labs in the USA began using enzyme-linked immunoassay (EIA). It can be automated and can analyze results objectively.

In 2009, Immuno-Mycologics (IMMY) invented a new cryptococcal antigen lateral flow immunoassay (LFA) for diagnosis of cryptococcal infection. As LA, it provides qualitative and semi-quantitative experimental results, and the problem of subjective judgment still persists. Despite of that, LFA has some advantages over the former two assays. It is a rapid diagnostic test as the reaction time is less than 10 minutes. LFA is stable at room temperature and has low requirement for laboratory equipment, demonstrating its usefulness as a point-of-care assay for diagnosis of cryptococcosis in developing countries.

Some studies have been conducted to evaluate the performance of these methods. As the results reported by different authors showed variance for the diagnostic role of different antigen detection methods, the real sensitivities and specificities of such methods remain unclear. Hence, we performed this meta-analysis aiming to systematically review all relevant studies to evaluate the diagnostic accuracy of the cryptococcal antigen LFA on serum, CSF and urine specimens.

## Materials and Methods

### Data Sources and Searches

Two investigators (HRH LCF) searched several public databases, including PubMed, Web of Science, Elsevier Science Direct and Cochrane Library for the English articles published till September 2014 respectively.”Cryptococcal antigen test”,”lateral flow assay”,”cryptococcal antigen lateral flow assay”,”LFA”,”cryptococcosis” and”diagnosis” were the key words. An expanded hand search of references of the relevant articles was also performed. We not only collected data from published full-text papers, but also from meeting and conference abstracts. We contacted the authors to obtain the unpublished data by e-mail.

### Study Selection

Two investigators (HRH LCF) first independently screened articles by title and abstract to produce a list of articles for full text review, then resolved the differences by discussion. We included studies of adult patients admitted to the hospital suspected with cryptococcosis (e.g. HIV-infected persons suspected with meningitis) that provided data that could be used to construct a two-by-two cross-tabulation for LFA against a reference test. All reference standards have blood culture and/or LA and/or EIA included. We excluded studies in which LFA was applied to samples other than serum, CSF and urine. We excluded reviews, duplicated studies which only described the LFA. Studies that reported unavailable data were excluded as well. If the same authors had published two or more studies using the same or overlapping dataset, only one study was included.

### Quality Assessment and Publication Bias

Two investigators (HRH LCF) assessed the risk of bias in each study by using the QUADAS-2 tool respectively [5]. Discrepancies were solved by discussion to get a consensus assessment and there was an adjudicator in case of persistent disagreement. We performed Deek’s funnel plot asymmetry test to detect publication bias [6].

### Data Extraction

Two investigators (HRH LCF) extracted the data independently using the pre-designed data extraction protocol. Extracted data included study details (e.g., the first author’s name, year of publication, design of studies, nationality, sample size, article type, etc) and the patient’s characteristics. We contacted authors of conference abstracts to obtain further information for clarification. Discrepancies were resolved by discussing or by contacting the original investigators. Data was extracted from studies directly, or estimated using common mathematical methods.

### Statistical Analysis

We calculated pooled estimates of the sensitivity, specificity and its 95% confidence interval (95% CI) for each study by using the DerSimonian and Laid method in the random effect model [7]. We also reported the positive and negative likelihood ratio (PLR and NLR) and the diagnostic odds ratio (DOR). The summary receiver operating characteristic (SROC) curve was used to represent the performance of the assay in each sample types. The area under the curve (AUC) and an index Q were discussed as potentially useful summaries of the curve [8]. We assessed statistical heterogeneity of the studies by the chi-square test, expressed with the I^2^ index [9]. A significant χ^2^ (p < 0.10) or I^2^-statistic (I^2^ > 50) indicated heterogeneity across studies. We performed threshold analysis and subgroup meta-analysis when heterogeneity was detected. Analyses were performed using the Meta-DiSc software v.1.4 and the Review Manager5.1software. All p values were two-sided and P<0.05 were considered statistically significant.

## Results

### Search Results

We had 719 potentially relevant papers from database search. After the duplicates were removed, there were 293 reports left. We excluded 261 irrelevant reports by reviewing abstract. With further screening of full texts, 19 studies were excluded for various reasons. At the end, we had 12 separate studies included in this meta-analysis. Detailed results of the literature search, selection, and reasons for exclusion are summarized in the flow diagram (S1 Fig).

### Characteristics of Eligible Studies

We identified three conference abstracts and nine full-length published articles till September 2014 [10–21]. Two abstracts and six full-length articles reported data on serum specimens. One abstract and five full-length articles included data on CSF specimens and three full-length articles included data on urine specimens. We included a total of 4622 specimens and the sample size ranged from 55 to 1000. We included prospective, retrospective, cross-sectional, cohort and case-control studies. The population evaluated in our meta-analysis consisted patients who were suspected for having cryptococcosis such as cryptococcal meningitis. A part of the populations was HIV-infected. The characteristics of the included studies are summarized in Table 1.

**Table 1:** Characteristics of studies included in the meta-analysis (n = 12).

Reference no.	First author	Year	Country	Article type	Sample size	Patient population	Study design	Sample type(s)
10	Jarvis	2011	SouthAfrica	Full article	62	HIV,CM	Retrospective case-control	Urine
11	Lindsley	2011	Thailand	Full article	551	HIV	Retrospective case-control	Serum Urine
12	Binnicker	2012	USA	Full article	683	SC	Retrospective case-control	Serum CSF
13	McMullan	2012	Australia	Full article	181	SC	Retrospective case-control	Serum Urine CSF
14	Clarke	2012	Australia	Conference abstracts	55	HIV	Retrospective cohort	Serum
15	Vijayan	2012	USA	Conference abstracts	197	HIV	Cross-sectional study	Serum
16	Rolfes	2012	Uganda	Conference abstracts	263	HIV,SC	Retrospective case-contro	CSF
17	Escandon	2013	Colombia	Full article	421	HIV	Retrospective case-control	Serum
18	Hansen	2013	USA	Full article	1000	SC	Prospective cohort	Serum CSF
19	Rugemalila	2013	Tanzania	Full article	319	HIV,SC	Cross-sectional study	Serum
20	Boulware	2014	SouthAfrica	Full article	666	HIV,SM	Retrospective case-control	CSF
21	Kabanda	2014	Uganda	Full article	224	SM	Prospective cohort	CSF

Abbreviations: HIV, hunman immunodeficiency virus; CM, cryptococcal meningitis; SC, suspected cryptococcosis; SM, suspected meningitis

### Risk of Bias Assessments

A summary of the risk of bias assessment results are shown in S2 Fig. Each of the 11 components according to QUADAS-2 criteria was graded as “yes”,”unclear” or “no”, which meant “low risk of bias”, “uncertain of bias” and “high risk of bias” respectively, based on the methods reported in each study. All studies met the requirement as an acceptable reference standard. In a number of studies, the assessment of risk of bias was affected by unclear reporting. Few studies implied blinding of the index test and reference test. Non-publication bias was detected by Deek’s funnel plot asymmetry test (P = 0.73).

### Data Synthesis and Meta-analysis

**Fig 1:**
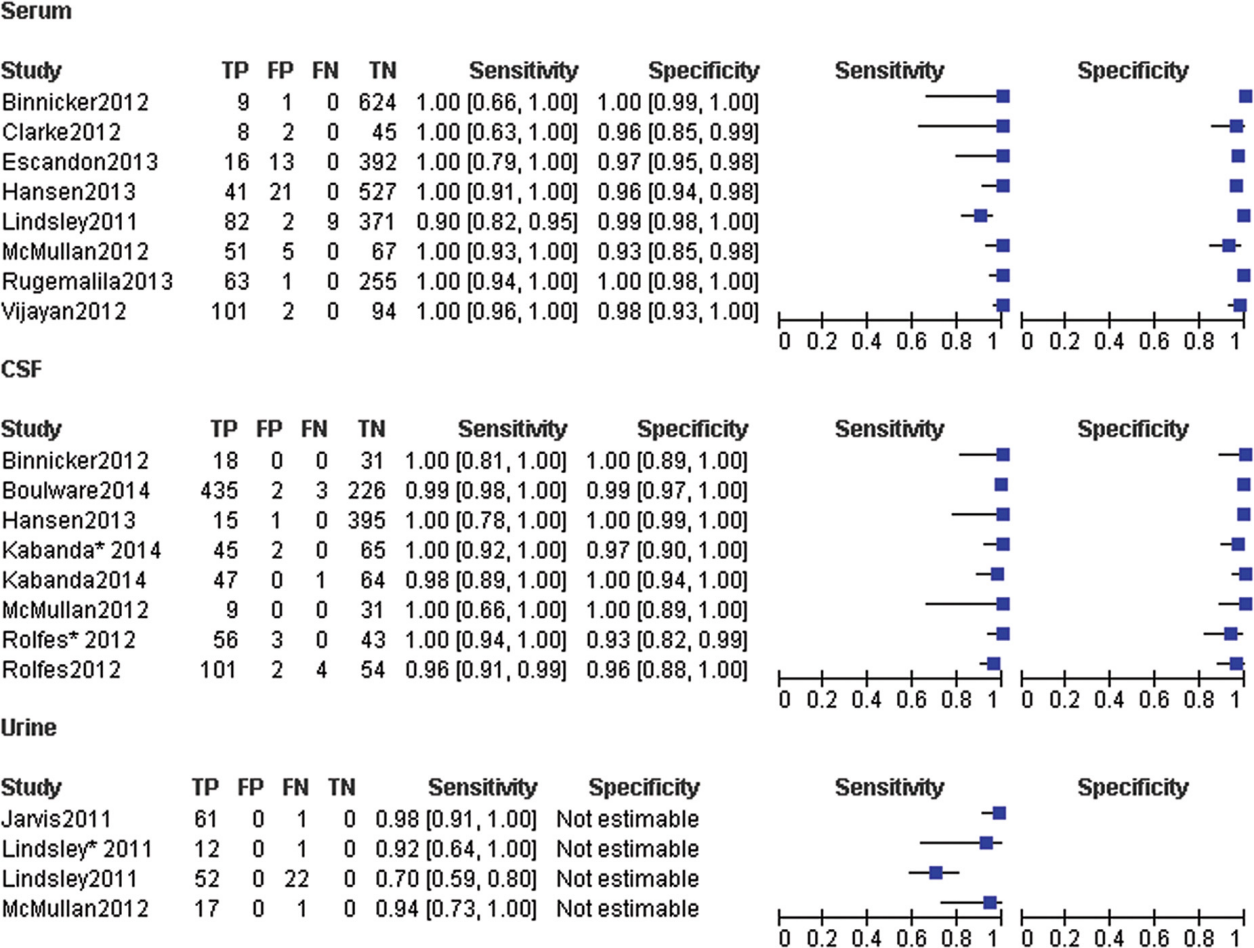
Forest plots of the sensitivity and specificity of LFA for the diagnosis of cryptococcal infection in serum, CSF and urine. Studies were classified into three categories according to the specimen types. Forest plots of sensitivity and specificity showed each study and the overall studies, respectively.

**Fig 2:**
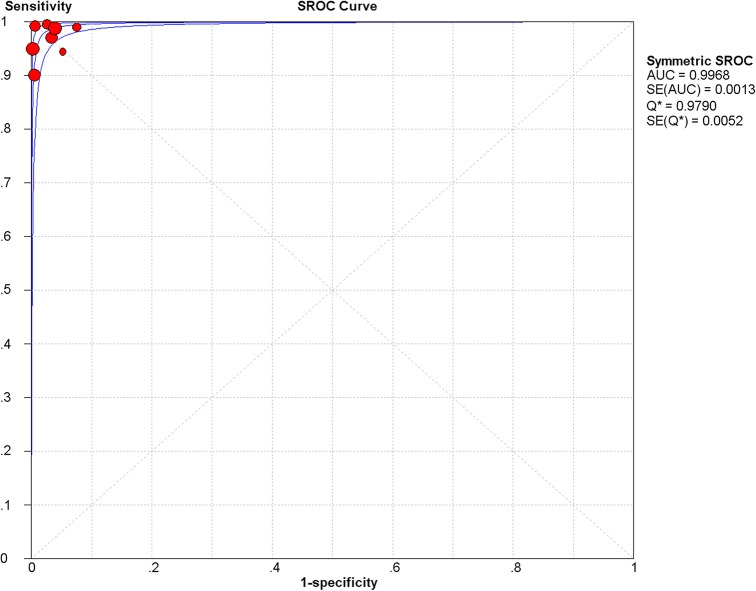
The summary receiver operating characteristic (SROC) curve of the LFA test in serum. Abbreviations: AUC, area under curve; SE, standard error; Q*, Q-statistic.

We classified studies into three categories according to the specimen types. Forest plots of sensitivity and specificity showed each study and the overall studies, respectively (Fig 1). For each study of serum specimens, sensitivity and specificity estimation with respect to the reference standard ranged from 90% to 100% and 93% to 100%, respectively. The pooled sensitivity and specificity values of the random effect model were 97.6% (95% CI, 95.6% to 98.9%) and 98.1% (95% CI, 97.4% to 98.6%), respectively (Table 2). The average PLR of LFA in serum was 43.79 (95% CI, 22.60–84.81) and the NLR was 0.03 (95% CI, 0.01–0.09). The pooled diagnostic odds ratio (DOR) was 2180.30 (95% CI, 868.92–5471.00) and the area under the curve (AUC) was 0.9968 (Fig 2).

**Table 2:** The diagnostic accuracy of LFA for cryptococcal infection in serum, CSF and urine.

Sample types	Parameter	Estimates	95%CI	χ^2^	P value	I^2^
Serum	Sensitivity	0.98	0.96–0.99	26.43	0.00	73.50%
	Specificity	0.98	0.97–0.99	45.32	0.00	84.60%
	Positive LR	43.79	22.60–84.81	29.90	0.00	76.60%
	NegativeLR	0.03	0.01–0.09	14.55	0.04	51.90%
	DOR	2180.30	868.92–5471.00	5.29	0.63	0.00%
CSF	Sensitivity	0.99	0.98–1.00	8.63	0.28	18.90%
	Specificity	0.99	0.98–1.00	16.00	0.03	56.20%
	Positive LR	48.83	21.59–110.40	14.07	0.05	50.30%
	NegativeLR	0.02	0.01–0.04	7.19	0.41	2.60%
	DOR	2931.10	1149.20–7475.90	7.16	0.41	2.20%
Urine	Sensitivity	0.85	0.79–0.90	25.93	0.00	88.40%

Abbreviations: CI, confidence interval; LR, likelihood ratio; DOR, diagnostic odds ratio.

**Fig 3:**
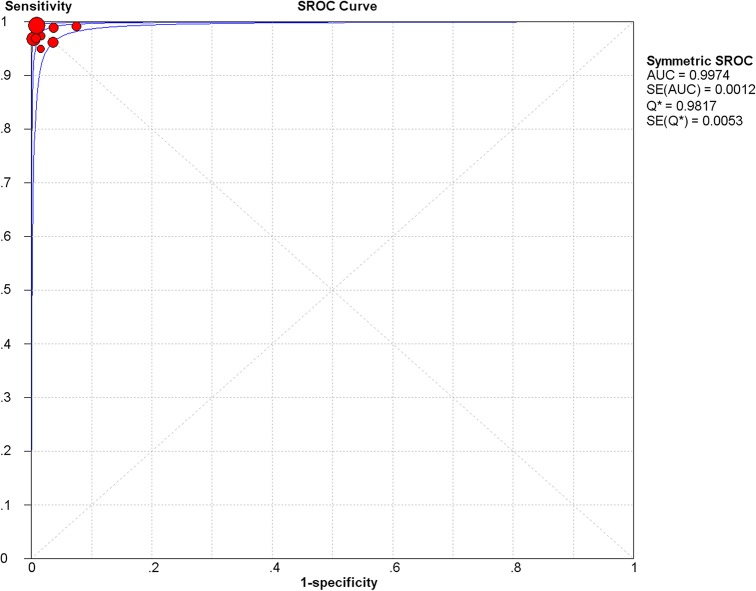
The summary receiver operating characteristic (SROC) curve of the LFA test in CSF. Abbreviations: AUC, area under curve; SE, standard error; Q*, Q-statistic.

For CSF specimens, sensitivity and specificity estimation with respect to the reference standard in each study ranged from 96% to 100% and 93% to 100%, respectively (Fig 1). The pooled sensitivity and specificity values of the random effect model were 98.9% (95% CI, 97.9% to 99.5%) and 98.9% (95% CI, 98.0% to 99.5%), respectively (Table 2). The average PLR of LFA in serum was 48.83 (95% CI, 21.59–110.40) and the NLR was 0.02 (95% CI, 0.01–0.04). The pooled diagnostic odds ratio (DOR) was 2931.10 (95% CI, 1149.20–7475.90) and the area under the curve (AUC) was 0.9974 (Fig 3).

For urine specimens, sensitivity estimation with respect to the reference standard in each study ranged from 70% to 98% (Fig 1). The pooled sensitivity value of the random effect model was 85.0% (95% CI, 78.7% to 90.1%) (Table 2).

We found significant heterogeneity for all the test performances because I-square values were above 50% except the sensitivity in CSF which was 18.90%. In subgroup analyses (Table 3), the sensitivity of subgroups were all above 90% and the specificity were all above 95% except the EIA reference subgroup in urine specimen. Its sensitivity was 83.1%. In the analysis of potential influence of heterogeneity, the I-square value of the EIA reference subgroups in serum and urine were both over 50%. Although the I-square value of the LA reference subgroup in CSF specimen was 50.2%, the sensitivity and specificity of it were 96.9% (95% CI, 92.9% to 99.0%) and 98.7% (95% CI, 95.3% to 99.8%), respectively, which were relatively high.

**Table 3:** Subgroup analyses of LFA for the diagnosis of cryptococcal infection in serum, CSF and urine.

Sample types	Subgroup study	Sensitivity	Specificity	χ^2^	P value	I^2^
Serum	LA	1.00[0.98–1.00]	0.98[0.97–0.99]	0.00	1.00	0.00%
	EIA	0.95[0.91–0.98]	0.98[0.97–0.99]	18.19	0.00	89.00%
CSF	LA	0.97[0.93–0.99]	0.99[0.95–1.00]	4.01	0.13	50.20%
	EIA	1.00[0.89–1.00]	1.00[0.99–1.00]	0.15	0.70	0.00%
	Culture	0.99[0.98–1.00]	0.98[0.96–0.99]	1.25	0.54	0.00%
Urine	EIA	0.83[0.76–0.89]	NA	23.32	0.00	95.70%
	Culture	0.94[0.79–0.99]	NA	0.06	0.81	0.00%

## Discussion

Many studies [10–21] have reported on the diagnostic value of the lateral flow immunoassay in detecting cryptococcal antigen. These studies, however, have demonstrated mixed results due to small sample sizes or low statistical power. In our meta-analysis, we combined 12 separate studies, consisting of 4622 samples to evaluate the diagnostic accuracy of the LFA for cryptococcal infection on serum, CSF and urine specimens. We found that the pooled sensitivity was 97.6% (95% CI, 95.6% to 98.9%) and the pooled specificity was 98.1% (95% CI, 97.4% to 98.6%) for serum specimen, which predicts LFA in serum is a very good method for diagnosis of cryptococcosis. For CSF specimen, the pooled sensitivity and specificity were both higher than those in serum. We speculated that the possible reason was because the majority of patients were suspected with cryptococcal meningitis. It also suggests us to conduct a lumbar puncture examination to identify cryptococcal infection on suspected patients. The pooled sensitivity of LFA in urine was 85.0% (95% CI, 78.7% to 90.1%). The pooled specificity was not estimated because of unavailable data. The sensitivity in urine was lower compared to that in serum or CSF. Nevertheless, urine specimen is easier to collect than cerebrospinal fluid, it provides us a convenient way to screen the suspected patients.

Subgroup analyses were performed for further description of the results. We found that the LFA was highly unanimous with the cryptococcal LA on serum specimens. However, in the EIA subgroup, the sensitivity was relatively lower than the specificity and the I-square value was 89.00%, which indicates significant heterogeneity. The performance of LFA was highly consistent with EIA and fungal culture method on CSF specimens. Although I-square value of the LA reference subgroup in CSF specimen was 50.2%, we still considered a little heterogeneity because of p>0.1. For urine specimens, there was heterogeneity between studies because of two different reference standards. The test performance of LFA was more consistent with culture than with EIA. When urine was evaluated, the LFA indicated higher sensitivity (93.5%) than EIA.

Our study had some limitations. Firstly, it is hard to ensure that no data was missed although we tried to collect all relevant papers. Secondly, we included only English articles and published papers which may lead to bias. Thirdly, significant between-study heterogeneity was detected in the current meta-analysis which may weaken conclusions’ reliance. Different diagnostic criteria may contribute to the heterogeneity among the selected studies. Despite the poor sensitivity, culture remains the gold standard one.

In summary, this meta-analysis demonstrates a very high accuracy of LFA in serum and CSF for the diagnosis of cryptococcosis for patients at risk. LFA in urine can be a promising sample screening tool for early diagnosis of cryptococcosis. Further studies testing CrAg in urine for diagnosis of cryptococcosis need to be performed.

## Supporting Information

S1 Text Checklist. (DOC)

S2 Text List of full-text excluded articles. (DOC)

S1 Fig Flow diagram of selection of studies for the meta-analysis. (TIF)

S2 Fig Summary of the methodological quality assessment of the included studies according to QUADAS-2 criteria. (TIF)
