# Supplementary material for: Evaluation of a New Cryptococcal Antigen Lateral Flow Immunoassay in Serum, Cerebrospinal Fluid and Urine for the Diagnosis of Cryptococcosis: A Meta-Analysis and Systematic Review
Source: PLoS One. 2015 May 14;10(5):e0127117. doi: 10.1371/journal.pone.0127117 (PMC4431798; doi:10.1371/journal.pone.0127117)
Supplement: S2 Text — (DOC) [file pone.0127117.s002.doc]

**List of full-text excluded articles and the reasons for exclusion**

There are 19 studies excluded by review of full text.

**6 reviews:**

1. Integrating cryptococcal antigen screening and pre-emptive treatment into routine HIV care. Rajasingham R, Meya DB, Boulware DR. J Acquir Immune Defic Syndr. 2012 Apr 15;59(5):e85-91.

2. Sensitivity and specificity of a new cryptococcal antigen lateral flow assay in serum and cerebrospinal fluid. Vijayan T, Chiller T, Klausner JD. MLO Med Lab Obs. 2013 Mar;45(3):16, 18, 20.

3. Frequency, diagnosis and management of fungal respiratory infections. Hayes GE, Denning DW. Curr Opin Pulm Med. 2013 May;19(3):259-65.

4. Fungal diagnosis: how do we do it and can we do better? Perfect JR. Curr Med Res Opin. 2013 Apr;29 Suppl 4:3-11.

5. Pulmonary fungal infections. Smith JA, Kauffman CA. Respirology. 2012 Aug;17(6):913-26.

6. Fungal central nervous system infections: prevalence and diagnosis. Kourbeti IS, Mylonakis E. Expert Rev Anti Infect Ther. 2014 Feb;12(2):265-73.

**5 for unsuitable reference standard:**

1. Use of the correlation coefficient to compare a point-of-care antigen test against a quantitative sandwich enzyme-linked immunosorbent assay for the detection of cryptococcal meningitis. Lee SJ, Newton PN. Clin Infect Dis. 2012 Dec;55(12):1744-5; author reply 1745-6.

2. Prevalence and correlates of cryptococcal antigen positivity among AIDS patients--United States, 1986-2012. McKenney J, Smith RM, Chiller TM, Detels R, French A, Margolick J, Klausner JD; Centers for Disease Control and Prevention. MMWR Morb Mortal Wkly Rep. 2014 Jul 11;63(27):585-7.

3. Prevalence of cryptococcal antigenemia and cost-effectiveness of a cryptococcal antigen screening program--Vietnam. Smith RM, Nguyen TA, Ha HT, Thang PH, Thuy C, Lien TX, Bui HT, Le TH, Struminger B, McConnell MS, Fanfair RN, Park BJ, Harris JR. PLoS One. 2013 Apr 23;8(4):e62213.

4. Serotype sensitivity of a lateral flow immunoassay for cryptococcal antigen. Gates-Hollingsworth MA, Kozel TR. Clin Vaccine Immunol. 2013 Apr;20(4):634-5.

5. Detection of antibody against fungal glucosylceramide in immunocompromised patients: a potential new diagnostic approach for cryptococcosis. Qureshi A, Wray D, Rhome R, Barry W, Del Poeta M. Mycopathologia. 2012 Jun;173(5-6):419-25.

**8 for unavailable data:**

1. Utility of urine and serum lateral flow assays to determine the prevalence and predictors of cryptococcal antigenemia in HIV-positive outpatients beginning antiretroviral therapy in Mwanza, Tanzania. Magambo KA, Kalluvya SE, Kapoor SW, Seni J, Chofle AA, Fitzgerald DW, Downs JA. J Int AIDS Soc. 2014 Aug 8

2. Significance of antibody detection in the diagnosis of cryptococcal meningitis. Patil SA, Katyayani S, Arvind N. J Immunoassay Immunochem. 2012;33(2):140-8.

3. Integrating cryptococcal antigen screening and pre-emptive treatment into routine HIV care. Rajasingham R, Meya DB, Boulware DR. J Acquir Immune Defic Syndr. 2012 Apr 15;59(5):e85-91.

4. False-positive cryptococcal antigen test associated with use of BBL Port-a-Cul transport vials. Wilson DA, Sholtis M, Parshall S, Hall GS, Procop GW. J Clin Microbiol. 2011 Feb;49(2):702-3.

5. Cryptococcal disease in patients with or without human immunodeficiency virus: clinical presentation and monitoring of serum cryptococcal antigen titers. Lin TY, Yeh KM, Lin JC, Wang NC, Peng MY, Chang FY. J Microbiol Immunol Infect. 2009 Jun;42(3):220-6.

6. Cost effectiveness of cryptococcal antigen screening as a strategy to prevent HIV-associated cryptococcal meningitis in South Africa. Jarvis JN, Harrison TS, Lawn SD, Meintjes G, Wood R, Cleary S. PLoS One. 2013 Jul 19;8(7):e69288.

7. The impact of routine cryptococcal antigen screening on survival among HIV-infected individuals with advanced immunosuppression in Kenya. Meyer AC, Kendi CK, Penner JA, Odhiambo N, Otieno B, Omondi E, Opiyo E, Bukusi EA, Cohen CR. Trop Med Int Health. 2013 Apr;18(4):495-503.

8. Significance of antibody detection in the diagnosis of cryptococcal meningitis. Patil SA, Katyayani S, Arvind N. J Immunoassay Immunochem. 2012;33(2):140-8.
