# Supplementary figures and images for: Evaluation of a New Cryptococcal Antigen Lateral Flow Immunoassay in Serum, Cerebrospinal Fluid and Urine for the Diagnosis of Cryptococcosis: A Meta-Analysis and Systematic Review
Source: PLoS One. 2015 May 14;10(5):e0127117. doi: 10.1371/journal.pone.0127117 (PMC4431798; doi:10.1371/journal.pone.0127117)

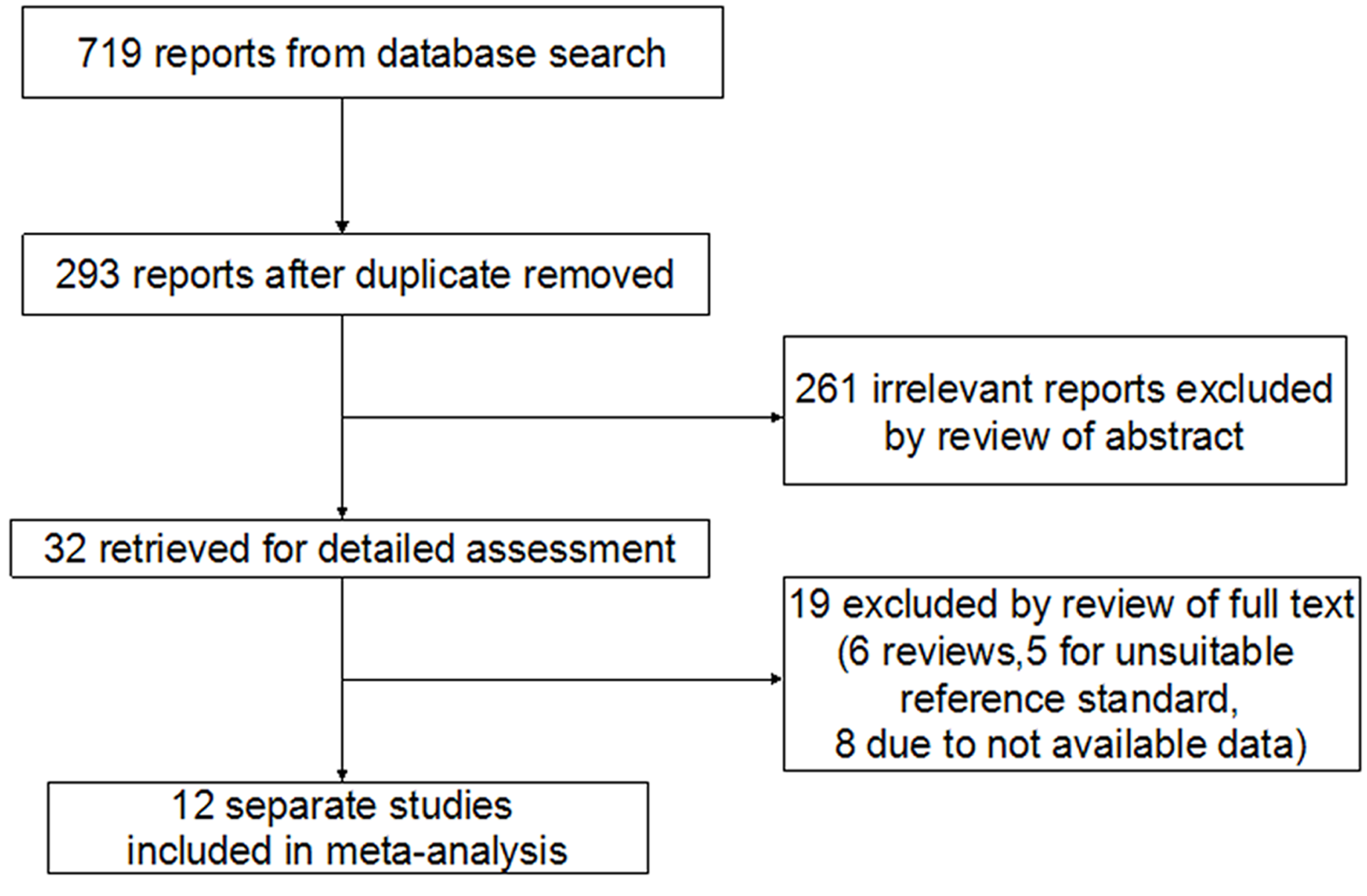

Supplement: S1 Fig — (TIF) [file pone.0127117.s003.tif]

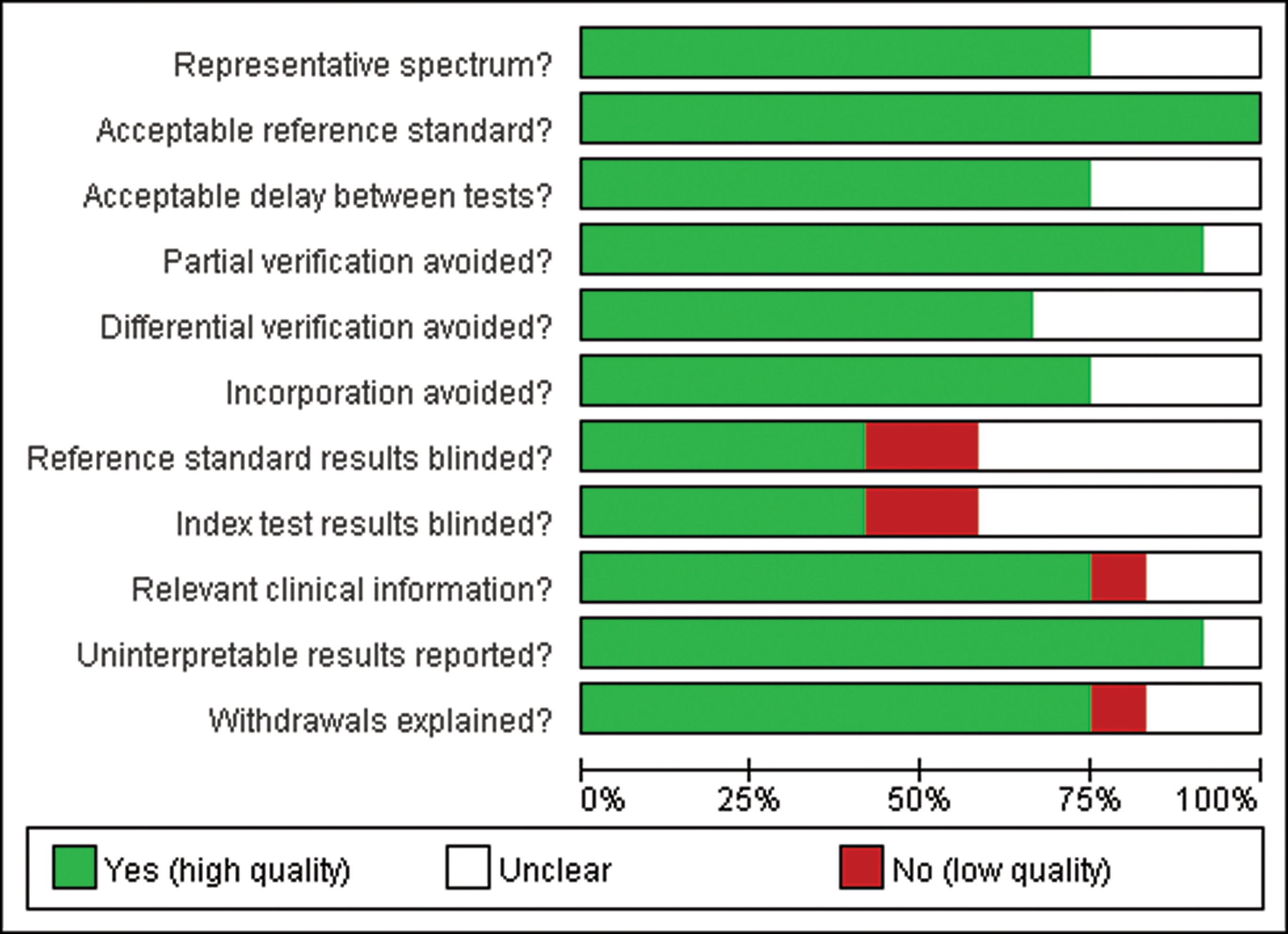

Supplement: S2 Fig — (TIF) [file pone.0127117.s004.tif]
